# Sapling growth rates reveal conspecific negative density dependence in a temperate forest

**DOI:** 10.1002/ece3.3298

**Published:** 2017-08-18

**Authors:** Benjamin S. Ramage, Daniel J. Johnson, Erika Gonzalez‐Akre, William J. McShea, Kristina J. Anderson‐Teixeira, Norman A. Bourg, Keith Clay

**Affiliations:** ^1^ Biology Department Randolph‐Macon College Ashland VA USA; ^2^ Earth and Environmental Sciences Division Los Alamos National Laboratory Los Alamos NM USA; ^3^ Conservation Ecology Center Smithsonian Conservation Biology Institute Front Royal VA USA; ^4^ Center for Tropical Forest Science Smithsonian Tropical Research Institute Panama City Panama; ^5^ U.S. Geological Survey National Research Program – Eastern Branch Reston VA USA; ^6^ Department of Biology Indiana University Bloomington IN USA

**Keywords:** Center for Tropical Forest Science—Forest Global Earth Observatory, Conspecific negative distance dependence, forest composition, forest dynamics, Janzen–Connell effects, neighborhood, plant–plant interactions, point pattern analysis, spatial analysis

## Abstract

Local tree species diversity is maintained in part by conspecific negative density dependence (CNDD). This pervasive mechanism occurs in a variety of forms and ecosystems, but research to date has been heavily skewed toward tree seedling survival in tropical forests. To evaluate CNDD more broadly, we investigated how sapling growth rates were affected by conspecific adult neighbors in a fully mapped 25.6 ha temperate deciduous forest. We examined growth rates as a function of the local adult tree neighborhood (via spatial autoregressive modeling) and compared the spatial positioning of faster‐growing and slower‐growing saplings with respect to adult conspecific and heterospecific trees (via bivariate point pattern analysis). In addition, to determine whether CNDD‐driven variation in growth rates leaves a corresponding spatial signal, we extended our point pattern analysis to a static, growth‐independent comparison of saplings and the next larger size class. We found that negative conspecific effects on sapling growth were most prevalent. Five of the nine species that were sufficiently abundant for analysis exhibited CNDD, while only one species showed evidence of a positive conspecific effect, and one or two species, depending on the analysis, displayed heterospecific effects. There was general agreement between the autoregressive models and the point pattern analyses based on sapling growth rates, but point pattern analyses based on single‐point‐in‐time size classes yielded results that differed markedly from the other two approaches. Our work adds to the growing body of evidence that CNDD is an important force in temperate forests, and demonstrates that this process extends to sapling growth rates. Further, our findings indicate that point pattern analyses based solely on size classes may fail to detect the process of interest (e.g., neighborhood‐driven variation in growth rates), in part due to the confounding of tree size and age.

## 1 INTRODUCTION

Species diversity in forests is maintained in part by conspecific negative density dependence (CNDD), a reduction in establishment, survival, and/or growth rates when conspecific densities are high, owing to the local accumulation of species‐specific natural enemies and/or intraspecific competitive interactions (Comita et al., 2014; Terborgh, 2012). Initial inquiries focused on diversity maintenance in species‐rich tropical forests (the Janzen–Connell Hypothesis; Connell, 1971; Janzen, 1970), and most subsequent research has examined seedling mortality in the tropics (e.g., Augspurger, 1983; Bagchi et al., 2014; Bell, Freckleton, & Lewis, 2006; Harms, Wright, Calderón, Hernández, & Herre, 2000; Wills, Condit, Foster, & Hubbell, 1997). However, a growing body of research suggests that proximate conspecific adults also reduce tree seedling survival in temperate forests (Bennett et al., 2017; Hille Ris Lambers, Clark, & Beckage, 2002; Johnson, Beaulieu, Bever, & Clay, 2012; Packer & Clay, 2000; Yamazaki, Iwamoto, & Seiwa, 2009).

Conspecific negative density dependence is a well‐documented driver of seed and seedling mortality for many species in many communities, but fewer studies have examined other measures of performance or other ontogenetic stages (Comita et al., 2014; Newbery & Stoll, 2013; Terborgh, 2012). One key variable, growth rate, could be particularly important for explaining the spatial patterns of larger individuals in size‐structured communities like forests. For instance, if adult trees disproportionately and severely reduce the growth of conspecific juveniles in their vicinity, this process could theoretically increase the local diversity of the canopy even without conspecific effects on juvenile survival. Furthermore, conspecific effects on growth rates have the potential to affect older individuals and/or larger size classes, because mortality beyond the seedling stage is usually little affected by local conspecifics (Wills & Condit, 1999; “Zhu et al. 2015a), although there are exceptions (e.g., Wang et al., 2012). Whether or not CNDD‐driven effects on growth rate also primarily affect seedlings remains unknown given the strong emphasis on survival in the literature (Comita et al., 2014; Newbery & Stoll, 2013). Reduced conspecific growth rates could potentially be driven by specialist natural enemies (e.g., Janzen–Connell effects), species‐specific resource needs that cause intraspecific competition to exceed interspecific competition (e.g., the Resource Ratio Hypothesis; Tilman, 1985), or by complex interactions that blend both of these mechanisms.

Despite the emphasis on seedling survival, several studies have examined the effects of conspecific neighbors on growth rates. In one of the earliest papers from a 50‐ha mapped tropical forest plot on Barro Colorado Island (BCI), Panama, Hubbell, Condit, Foster, Grubb, and Thomas (1990) found a 23.5% reduction in annual growth of stems 1–4 cm in diameter, but only a 5.3% reduction in survival, in the vicinity of conspecific versus heterospecific adults. In a later study at BCI, Condit, Hubbell, and Foster (1994) examined an abundant understory tree species, *Faramea occidentalis*, and found that small individuals grew more slowly when large conspecific neighbors were nearby. In a mapped tropical forest plot in Borneo, Newbery and Stoll (2013) found that most overstory species showed strong negative effects on conspecific stem growth rates, but these patterns were weaker in a second census following an El Nino ENSO event. Finally, in a study of *Microberlinia bisulcata* from a tropical forest in Cameroon, Norghauer and Newbery (2016) found that growth rates decreased with increasing conspecific adult basal area for some but not all juvenile classes.

In temperate forests, there is also evidence of differential effects of conspecific versus heterospecific neighbors on growth rates. However, in temperate ecosystems, general intra‐ versus interspecific effects have received less emphasis than species‐specific competition coefficients, and the intended application for this research has often been applied forest management models (e.g., SORTIE) rather than attempts to explain biodiversity (e.g., Canham et al., 2006). This distinction arises in part because the lower number of species in temperate forests facilitates a species‐specific approach. While the greater detail provided by a species‐specific approach may seem preferable in some situations, it also deflects attention from CNDD‐driven biodiversity maintenance, which depends chiefly on the distinction between conspecific and heterospecific effects. Nevertheless, previous studies have documented differential effects of conspecific and heterospecific neighbors. For instance, Canham et al. (2006) examined the effects of competition on the growth of the 14 most abundant tree species in New England forests as a function of target tree size, crowding by neighboring trees, and environmental conditions. For many species, the relative strength of intraspecific competition was greater than interspecific competition, and for 13 of 14 species, there was no support for the hypothesis that all species had equivalent competitive effects. In the temperate cedar‐hemlock forests of British Columbia, Coates, Canham, and LePage (2009) examined competitive effects and responses among nine co‐occurring tree species as a function of tree size and distance. They found limited evidence of CNDD for less common species, but clear evidence that the most abundant species, *Tsuga heterophylla*, was more strongly inhibited by conspecifics.

There is also a parallel literature on tree performance in “pure” (monoculture) versus “mixed” (polyculture) stands (e.g., Cavard et al., 2011; Lilles & Coates, 2014). This body of research differs from the above approach in that spatially explicit neighborhood relations are generally not considered; the emphasis is instead on stand‐level summary data (e.g., total biomass accumulation) (Perot, Goreaud, Ginisty, & Dhôte, 2010). In addition, the focus is often on planted forests, not naturally regenerated forests, and thus, biodiversity maintenance is rarely of interest. As an example, *Fagus sylvatica* in European forests exhibited lower growth rates and drought resistance in pure stands than in mixed stands (Mölder & Leuschner, 2014), suggesting that conspecific neighbors inhibit plant performance. However, there is no clear consensus on whether pure or mixed stands are more productive (Lilles & Coates, 2014; Lübbe, Schuldt, & Leuschner, 2015).

If large conspecific neighbors negatively affect juvenile growth (with or without effects on survival), these effects could lead to characteristic spatial patterns. For instance, larger juvenile size classes (e.g., 5‐ to 10‐cm‐diameter stems) may typically occur farther from mature conspecific trees than smaller size classes (e.g., 1‐ to 5‐cm‐diameter stems). In support, Packer and Clay (2003) showed that biomass of naturally established *Prunus serotina* saplings was significantly higher for individuals farther away from adult conspecifics. Despite these findings, in most natural forests, spatial signatures like this may often be overwhelmed by several confounding factors (McIntire & Fajardo, 2009), including variation in establishment times (e.g., juveniles of variable and unknown age) and the mortality (and subsequent decay and disappearance) of trees that had a pronounced effect in the past. Nonetheless, spatial approaches may still have potential in complex stands. For instance, if growth rates are known, and juveniles of a given species and size class are separated into different growth rate classes, point pattern analysis could be used to determine whether slower‐growing individuals are disproportionately clustered around conspecific adults.

To investigate whether juvenile growth is affected by conspecific adult neighbors, we: (1) examined growth rates of *saplings* (trees 1–5 cm in diameter; Zhu et al. 2015a) as a function of their neighborhood, (2) analyzed spatial patterns of faster‐growing and slower‐growing saplings with respect to mature trees (conspecific and heterospecific), (3) conducted similar spatial pattern analyses but with groups based on size classes instead of growth rates, and (4) assessed the extent to which the results for each species corresponded across different analyses. Our results help to evaluate the extent to which CNDD occurs in temperate forests and extends beyond seedling mortality. In addition, our combination of methods (neighborhood models and point pattern analyses) establishes a framework for future analyses that could be applied to other datasets.

## 2 MATERIALS AND METHODS

### 2.1 Study site and data collection

We analyzed data collected at the Center for Tropical Forest Science—Forest Global Earth Observatory large forest dynamics plot located at the Smithsonian Conservation Biology Institute (SCBI) in Virginia, USA (38°53′36.6′′N, 78°08′43.4′′W). This is a 25.6‐ha plot (400 × 640 m) with elevation ranging from 273 to 338 m.a.s.l. (Bourg, McShea, Thompson, McGarvey, & Shen, 2013). It is located in a mature, mixed deciduous forest that developed after agricultural use in the mid‐19th century. Mean annual temperature from 2009 to 2014 was 12.9°C and mean annual precipitation was 1,001 mm, based on a nearby weather station (Anderson‐Teixeira et al., 2015). The dominant canopy tree species are *Liriodendron tulipifera* (tulip poplar), *Carya* spp. (hickories; 4 species)*, Quercus* spp. (oaks, 4 species), *Fraxinus americana* (white ash), and *Nyssa sylvatica* (black gum). A total of 46 tree species have been identified in the plot, seven of which are small‐statured species generally confined to the understory. In 2008 and 2013, tree censuses were conducted following standard CTFS‐ForestGEO protocols (Bourg et al., 2013; Condit, 1998) where all stems of diameter at breast height (DBH) ≥1 cm were mapped, tagged, identified to species, and measured for DBH. Four hectares within the plot have been fenced since 1990 to exclude *Odocoileus virginianus* (white‐tailed deer), which may have led to taller tree seedlings and greater sapling density within the exclosure (McGarvey, Bourg, Thompson, McShea, & Shen, 2013). All analyses reported in the main text include the area within the deer exclosure because: (1) this provides a larger sample size and enables analysis of a greater number of species, and (2) inclusion or omission of the deer exclosure led to only minor differences that did not affect our findings. A parallel set of analyses with the exclosure omitted is provided in the Supporting Information (Table S1, Fig. S1, Fig. S2). We did not conduct separate analyses of the area within the exclosure because direct comparisons of results inside and outside of the exclosure would be confounded by major differences in sample size, area, and shape.

### 2.2 Focal species and size classes

We examined only species that are capable of reaching canopy status and that had sufficient abundance in the plot for robust analysis (“common canopy species”; Table 1). We limited our analysis to canopy species for two reasons: (1) We required a clear size‐based distinction between adults and juveniles, and (2) species that are often multistemmed (e.g., shrubs or small stature trees) are problematic when testing for CNDD because stems with a common root system share resources and thus are not independent (none of our focal species are frequently multistemmed). We focused our analyses primarily on stems 1–5 cm DBH, hereafter called “saplings” (matching Zhu et al. 2015a), which are the smallest mapped stems at our study site. This decision was based on the presumption that if growth is to be affected by the local neighborhood, effects are likely to be stronger on smaller size classes. Due to the strong size‐asymmetric nature of interactions in mixed‐age forests (Schwinning & Weiner, 1998), smaller stems are more sensitive to competition and more susceptible to many natural enemies than larger individuals. Secondary stems (all stems other than the largest within a multistemmed genet) were not considered as focal trees because growth rates are likely to be affected by subsidies from larger ramets. Therefore, secondary stems within the sapling size class (approx. 8% of all saplings) were omitted. However, secondary stems were included in neighborhood basal area (BA) calculations (see below), as all stems in clumps of large trees were assumed to have additive influence. Dead stems (as of the initial 2008 survey) were excluded from consideration.

**Table 1:** Summary data for common canopy tree species

Species	Frequency by diameter class (cm)					Total BA in plot (m^2^/ha)	Median sapling growth rate (mm/yr)
	1–5	5–10	10–15	15–20	20+		
*Acer rubrum*	64	107	69	39	67	0.36	0.46
*Carya cordiformis*	114	123	53	45	110	0.60	0.84
*Carya glabra*	512	531	253	130	343	1.62	0.30
*Carya ovalis*	53	105	87	55	143	0.62	0.42
*Carya tomentosa*	394	392	219	132	240	1.06	0.38
*Fagus grandifolia*	67	170	139	81	49	0.42	0.98
*Fraxinus americana*	116	187	124	52	223	1.69	0.72
*Liriodendron tulipifera*	49	119	231	251	1,461	13.87	0.92
*Nyssa sylvatica*	390	466	219	127	139	0.72	0.24
All species in plot	17,625	4,643	2,141	1,221	4,394	33.34	0.90

Dead stems and secondary stems were excluded from stem counts and growth rate calculations, but total BA includes secondary stems. Calculations are based on 2008 data from the entire study site, including the deer exclosure. For comparison, the final row provides totals across all species, including those that were not individually analyzed. Oaks (*Quercus* spp.) accounted for the majority of the remaining BA (9.54 m^2^/ha), but oak saplings were rare (37 stems total across four different oak species), precluding analysis of sapling growth rates.

BA, basal area.

### 2.3 Regression analyses (including spatial autoregressive modeling)

We used multiple regression to determine the effect of the local neighborhood on sapling growth rate (see Newbery & Stoll, 2013 for a similar approach). The local neighborhood was quantified as the inverse distance‐weighted basal area (IDW BA) of adult conspecific and heterospecific neighbors. Adult neighbors consisted of trees ≥10 cm DBH, up to 15 m from the focal sapling, as several papers have found that conspecific effects disappear at approximately this distance (e.g., Hubbell, Ahumada, Condit, & Foster, 2001; Johnson et al., 2014). Accordingly, all saplings within 15 m of the plot boundary were excluded. Note that our usage of “adult” is strictly size‐based and thus does not necessarily indicate reproductive maturity, matching many similar papers (e.g., Zhu et al. 2015a). Annual growth rate was calculated as 2013 DBH minus 2008 DBH, divided by five. There was no relationship between initial size (2008 DBH) and annual growth rate for any of the species considered, so initial size was not included as a covariate. Annual growth rates were log‐transformed prior to analysis, yielding models with normally distributed and homoscedastic residuals.

To account for the possibility of spatial autocorrelation in sapling growth rates due to local differences in soil fertility or other undocumented variables, we used spatial autoregressive modeling (function *spautolm* in the *spdep* R package). These analyses included the same predictor variables (conspecific and heterospecific IDW BA), while also modeling residual spatial structure in sapling neighborhoods (neighbors were defined as all saplings within 15 m, and neighbor weights were inversely weighted by distance). Most species exhibited no residual spatial trends in growth rates after accounting for conspecific and heterospecific IDW BA, nullifying the need to account for spatial autocorrelation. For these species, results are from standard multiple regressions. For all species that did exhibit residual spatial structure in sapling growth rates, reported results are from spatial autoregressive models (but for convenience, we hereafter refer to all of these models as simply “regressions”). In addition, because several of the species in our dataset are *Carya* species, and there is evidence that the negative effects of closely related neighbors can extend beyond the species level and may be greater at later life stages (Zhu et al. 2015a), we used similar regression models to investigate the possibility of con*generic* NDD among *Carya* species. Detailed methods and the associated results are presented in the Supporting Information. All analyses were conducted in R version 3.2.1 (R Core Team 2015).

### 2.4 Point pattern analysis

We conducted bivariate point pattern analyses aimed at further exploring relationships between adult neighborhood composition and sapling growth rates. These analyses examine spatial patterns across a range of distances (e.g., clustering of small stems at different distances around adult conspecifics), and thus, they are technically a test of conspecific negative *distance* dependence, as opposed to conspecific negative *density* dependence (Comita et al., 2014). However, distance dependence and density dependence are often closely linked and mechanistically indistinguishable (Packer & Clay, 2000). Accordingly, like several recent papers (e.g., Johnson et al., 2014), we have adopted a broader definition of CN*density*D that encompasses distance‐dependent effects and effectively treats CN*distance*D as a special case of CN*density*D. For these analyses, we only considered the five species with sufficient abundance in all size classes analyzed: *Carya cordiformis*,* Carya glabra*,* Carya tomentosa*,* F. americana*, and *N. sylvatica* (Table 1). Illian, Penttinen, Stoyan, and Stoyan (2008) recommend a minimum of 70 points for robust point pattern analysis, and our preliminary analyses revealed unstable results for the other, less common species (i.e., high sensitivity to minor parameter adjustments).

Two different sets of bivariate point pattern analyses were conducted, both of which examined the clustering of small stems around both conspecific and heterospecific adults. Two distinct small stem groups were examined (see below), but adults in these analyses were consistently defined as trees ≥20 cm DBH. This large minimum value, relative to the 10 cm DBH threshold for adult trees used in the regressions, was selected for consistency with point pattern analyses in similar studies (e.g., Ramage & Mangana, 2017; Wiegand, Gunatilleke, Gunatilleke, & Okuda, 2007) and to minimize the chance of any given pair of small stem and adult points belonging to the same cohort. In the first set (termed “growth‐based point pattern analyses”), saplings (1–5 cm DBH) of each species were divided into *faster‐* and *slower*‐growing groups (individuals with growth rates above and below the median growth rate, respectively, for each species). These point pattern analyses are essentially asking the same question as the regressions (“is sapling growth rate affected by conspecific or heterospecific adult neighbors?”), but using different methods. If similar results are obtained with both approaches, it would confirm that the findings are robust. In the second set of bivariate point pattern analyses (termed “size‐based point pattern analyses”), we compared the clustering patterns of two small stem size classes: 1‐ to 5‐cm DBH stems (“1–5” hereafter, defined as “saplings” above) and 5‐ to 10‐cm DBH stems (“5–10” hereafter); note that we use the umbrella term “small stem” to encompass two size classes (1–5 and 5–10 cm DBH), while we apply the term “sapling” only to individuals 1–5 cm DBH. These size classes were chosen to match those used by Zhu et al. (2015a) and Wiegand et al. (2007) for similar analyses. For all species subjected to point pattern analysis, the number of small stems in each of the two size classes (1–5 and 5–10) was approximately equal (Table 1), and the size distribution between 1 and 10 cm DBH was roughly uniform.

Aside from the differences described above, all bivariate point pattern analysis details were identical for analyses based on growth rate and by size class. For each focal species, conspecific and heterospecific adults were considered separately, and heterospecific adults of all species were pooled together. In all cases, inhomogeneous bivariate pair correlation functions were used (function *pcfcross.inhom* in the *spatstat* R package); inhomogeneous functions relax the assumption of spatial homogeneity and allow the intensity of the point process to vary across space. For each focal species, an inhomogeneous surface was created separately for conspecific adults and heterospecific adults, but then a single surface was used for small stems (faster and slower combined in the growth‐based analyses, or 1–5 and 5–10 combined in the size‐based analyses). This accounts for spatial heterogeneity in small stem density while treating the small stem classes equivalently (matching our null models described below).

The main focus of our point pattern analyses was not on how small stems were positioned around adult conspecific or heterospecific trees, but rather on *differences* in spatial patterning *between* small stem groups (fast vs. slow, or 1–5 vs. 5–10). To determine the *significance of the difference* between small stem groups, we used an approach similar to Larson et al. (2015), including a comparable null model (“random labeling”). We repeated the following steps 1000 times to generate null distributions for our bivariate pair correlation functions: (1) shuffle labels across small stem groups (“fast”/“slow” status across focal saplings in the growth‐based analyses, or “1–5”/ “5–10” status across focal small stems in the size‐based analyses), (2) calculate the bivariate pair correlation function for each small stem group, and (3) subtract one set of values from the other (slow minus fast, or 1–5 minus 5–10). Next, we calculated a 95% confidence envelope for the simulated *differences* between groups (2.5th through 97.5th percentile), and determined the distance bands for which the actual difference between small stem groups (slow minus fast, or 1–5 minus 5–10) departed from the confidence envelope. This null model assumes that each pair of small stem groups was originally undifferentiated and that group classification (e.g., “fast” or “slow”) was unaffected by proximity to adult trees.

## 3 RESULTS

Effects of the local adult neighborhood on sapling growth rates were variable, with negative conspecific effects most common (Table 2). Significant negative conspecific effects were detected for five of nine species (*Fagus grandifolia* and all four *Carya* species), while positive conspecific effects were found for only one (*L. tulipifera*). However, partial *r* ^*2*^ values were low for all conspecific effects (ranging from approximately .02 to .10), suggesting that most of the variation in growth rate is driven by other factors. By contrast, the partial *r* ^*2*^ associated with the positive conspecific effect for *L. tulipifera* was fairly high (.289). The three remaining species (*A. rubrum, F. americana*, and *N. sylvatica*) showed no significant conspecific effects. Significant heterospecific effects were detected for only two of nine species (*C. cordiformis* and *C. tomentosa*), and both were negative. For all species except *L. tulipifera,* the vast majority of IDW BA was heterospecific (Table 2), and thus, the negative conspecific effects we detected are definitively linked to conspecific abundance, as opposed to simply reflecting a response to higher total basal area. Models for the following species incorporated significant (or borderline significant) spatial autocorrelation effects: *C. glabra* (*p *< .001), *F. grandifolia* (*p *= .066), and *N. sylvatica* (*p* = .001). All other species had nonsignificant residual spatial structure in growth rates after accounting for the main effects.

**Table 2:** Sapling growth rate as a function of conspecific and heterospecific inverse distance‐weighted basal area

Species	*n*	Conspecific effect				Heterospecific effect			
		*p*‐value	Est.	Partial *r* ^*2*^	Mean (±*SD*) con. IDW BA (m^2^)	*p*‐value	Est.	Partial *r* ^*2*^	Mean (±*SD*) het. IDW BA (m^2^)
*Acer rubrum*	45	.483	0.042	0.016	0.002 ± 0.004	.478	0.042	0.017	0.259 ± 0.070
Carya cordiformis*	104	.005	−0.250	0.099	0.006 ± 0.013	.002	−0.163	0.121	0.266 ± 0.112
Carya glabra*	462	.012	−0.049	0.021	0.015 ± 0.016	.594	−0.010	0.004	0.239 ± 0.078
*Carya ovalis*	51	.051	−0.086	0.089	0.008 ± 0.011	.916	−0.005	0.000	0.279 ± 0.082
Carya tomentosa *	368	.006	−0.054	0.023	0.013 ± 0.013	.049	−0.039	0.012	0.249 ± 0.079
*Fagus grandifolia*	60	.049	−0.170	0.073	0.034 ± 0.056	.796	−0.019	0.001	0.231 ± 0.100
Fraxinus americana*	90	.343	0.061	0.014	0.002 ± 0.004	.267	−0.051	0.019	0.259 ± 0.083
*Liriodendron tulipifera*	34	.004	0.302	0.289	0.151 ± 0.129	.221	0.118	0.059	0.117 ± 0.076
Nyssa sylvatica*	349	.564	0.012	0.006	0.012 ± 0.013	.396	−0.018	0.000	0.248 ± 0.071

Each sapling species was analyzed in a separate model, and for each model, both predictor variables were standardized so estimates are directly comparable across species and variables (slope estimates represent the predicted change in growth rate with one standard deviation increase in IDW BA). Partial *r* ^*2*^ values for conspecific and heterospecific effects are also provided. The mean (conspecific or heterospecific) IDW BA column displays the average predictor variable values for each sapling included in the analysis. Each row represents a single model (i.e., conspecific and heterospecific effects for each focal sapling species were analyzed concurrently). Sample sizes (“*n*”) do not match counts in the first column of Table 1 because saplings within 15 m of the plot edge were excluded. Species with asterisks are those for which point pattern analyses were conducted. A corresponding table for analyses omitting the deer exclosure is provided in the Supporting Information (Table S1).

BA, basal area; IDW, inverse distance‐weighted basal area.

**Figure 1:**
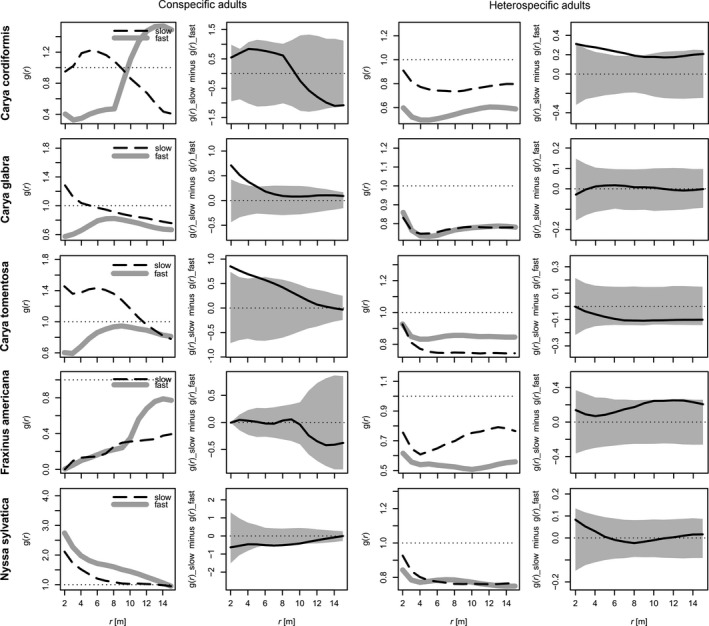
Spatial patterns of two sapling categories (slow‐ and fast‐growing) with respect to adult trees. Vertical axes indicate bivariate pair correlation function values (higher values represent increased clustering), and horizontal axes indicate distance (r) from adult trees. For both conspecific adults (left columns) and heterospecific adults (right columns), the panels with confidence envelopes display “slow” values minus “fast” values (the dashed black line minus the thick grey line); extensions above the envelope indicate that slow‐growing saplings are more clustered than fast‐growing saplings around adults (conspecific or heterospecific), and vice versa

The growth‐based point pattern analysis results (Figure 1) generally aligned with the results from our regressions (Table 2). We found that faster‐growing saplings of *C. glabra* and *C. tomentosa* were generally farther away from conspecific adults than slower‐growing saplings were (at distances up to approx. 5 m), which is consistent with the regression results. For *C. cordiformis*, the difference was not significant (i.e., the observed values were wholly within the confidence envelope), but the general pattern (faster‐growing saplings farther from adult conspecifics) was consistent with the regressions. Conspecific patterns for *F. americana* and *N. sylvatica* also mostly matched the regressions (where all effects were nonsignificant; Table 2), although the *N. sylvatica* point pattern results indicated a very slight positive conspecific association at distances around 8 m. The *F. americana* point pattern results revealed that most saplings, regardless of growth rate, were dispersed far from adult conspecifics (and there is no analogue to this in the regressions for comparison).

Heterospecific effects were also similar across the growth‐based point pattern analyses and regressions. Growth‐based point pattern results for *C. glabra*,* N. sylvatica*,* F. americana,* and *C. cordiformis* generally aligned with the regressions. The first three exhibited nonsignificant heterospecific effects in both the regressions and point pattern analyses, and *C. cordiformis* displayed evidence of a negative heterospecific effect in both. *C. tomentosa* exhibited a negative heterospecific effect in the regression, but no significant effects in the point pattern analysis, which is not surprising given the very low partial *r* ^*2*^ value (.012) for the heterospecific effect in the regression.

**Figure 2:**
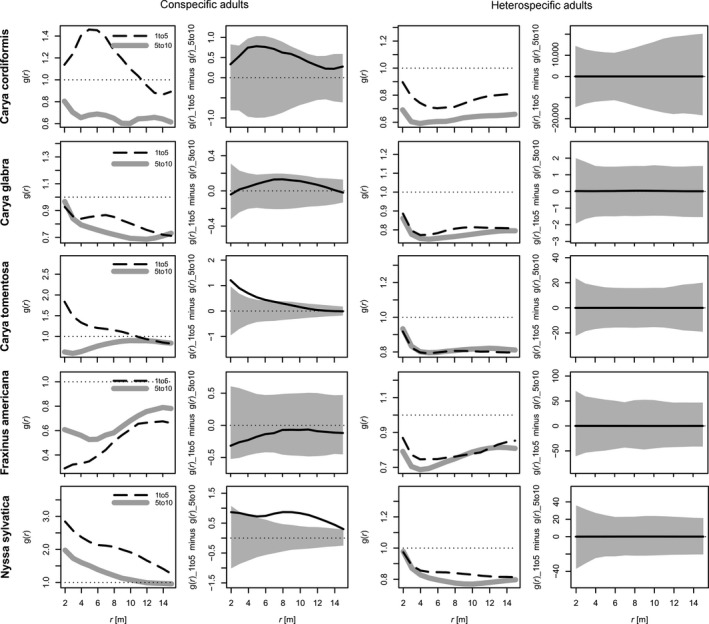
Spatial patterns of two small stem categories (1–5 cm diameter at breast height [DBH] [“saplings”] and 5–10 cm DBH) with respect to adult trees. Vertical axes indicate bivariate pair correlation function values (higher values represent increased clustering), and horizontal axes indicate distance (r) from adult trees. For both conspecific adults (left columns) and heterospecific adults (right columns), the panels with confidence envelopes display “1–5” values minus “5–10” values (the dashed black line minus the thick grey line); extensions above the envelope indicate that 1‐ to 5‐cm DBH stems are more clustered than 5‐ to 10‐cm DBH stems around adults (conspecific or heterospecific), and vice versa

Results from the size‐based point pattern analyses (Figure 2) were poorly aligned with results from both the regressions (Table 2) and the growth‐based point pattern analyses (Figure 1). The only significant effect that was consistent across the size‐based and growth‐based point pattern analyses was for *C. tomentosa*. In the size‐based analyses for this species, there was a significant trend of greater clustering around conspecific adults by 1‐ to 5‐cm DBH stems as compared to 5‐ to 10‐cm DBH stems, and there was a comparable trend of slow‐growing saplings clustered around conspecific adults in the growth‐based analyses. This same effect was also revealed by the regressions. In contrast, the conspecific results for *N. sylvatica* diverged substantially from both the growth‐based point pattern analyses and the regressions. In the size‐based analyses, 1‐ to 5‐cm DBH stems were significantly more clustered, as compared to 5‐ to 10‐cm DBH stems, around conspecific adults at intermediate distances. However, no evidence of conspecific inhibition in *N. sylvatica* was provided by either of the other two approaches. All other relationships, both conspecific and heterospecific, were nonsignificant in the size‐based point pattern analyses.

## 4 DISCUSSION

We found that negative conspecific effects were more common than positive conspecific effects or heterospecific effects of any type, providing further evidence that CNDD plays an important role in temperate forests. In addition, our focus on sapling growth rate demonstrates that CNDD extends well beyond its traditional domain of seedling mortality. Further, despite very different methodological approaches, results from our growth‐based point pattern analyses generally matched results from our regressions. As such, we can infer the action of CNDD with high confidence for particular species. For instance, saplings of two hickory species (*C. glabra* and *C. tomentosa*) consistently exhibited a reduction in growth rates when large conspecifics were nearby, and a similar pattern was found for *C. cordiformis* (although the effect was not significant in the growth‐based point pattern analyses, possibly due to a much smaller sample size). In contrast, neither our regressions nor our growth‐based point pattern analyses revealed any evidence of CNDD for *N. sylvatica*.

Our results align remarkably well with those of several related studies in terms of broad findings and species‐specific results. Three recent papers used observational approaches to compare conspecific and heterospecific effects across tree species in the eastern United States (Johnson et al., 2012; LaManna, Walton, Turner, & Myers, 2016; Zhu et al. 2015b), and all found that negative conspecific effects were more common and/or stronger than positive conspecific effects or any type of heterospecific effect. LaManna et al. (2016) assessed CNDD in saplings and found similar results for five species that were also included here, despite different methods (associations between sapling density and adult density) and a different study region (Missouri). Specifically, they found stronger CNDD for *C. glabra, C. tomentosa*, and *C. cordiformis* than for *A. rubrum* and *F. americana*. Zhu et al. (2015b) examined seedling‐to‐sapling recruitment in a dataset that spans the entire eastern United States and found results strikingly similar to ours for all overlapping species: negative conspecific effects for *C. glabra* and *F. grandifolia*, positive conspecific effects for *L. tulipifera*, and nonsignificant conspecific effects for *A. rubrum* and *N. sylvatica* (all based on adult BA; species‐specific results provided in their supplementary material). In contrast, the results in Johnson et al. (2012), which utilized an older version of the same dataset used by Zhu et al. (2015b), bear less resemblance to ours on a species‐by‐species basis. This may be because the analyses in Zhu et al. (2015b) are more methodologically similar to ours. Johnson et al. (2012) assessed static co‐occurrence patterns between seedlings and trees, while Zhu et al. (2015b) indirectly incorporated growth rates by assessing recruitment, and also extended their analyses beyond the seedling stage, which is important given that the strength of CNDD is affected by ontogeny (LaManna et al., 2016; Zhu et al. 2015a).

Canham et al. (2006) also studied species‐specific effects on growth rates, but focused on larger size classes (stems ≥12.7 cm DBH) and did not directly compare conspecific and heterospecific effects. In general agreement with our results, they found that *F. grandifolia* growth was strongly inhibited by conspecifics and that neighbor effects on *A. rubrum* were similar for conspecifics and other species. The only other overlapping species, *F. americana*, was more inhibited by conspecifics than by any other species. Neither of our analyses of *F. americana* (regressions or point pattern analyses) align with this finding, perhaps due to differences in size classes or study region (Mid‐Atlantic vs. New England). However, our results do reveal that most *F. americana* saplings, regardless of growth rate, occur far away from adult conspecifics, implying strong survival‐driven CNDD prior to the sapling stage. In agreement with this, LaManna et al. (2016) found very strong CNDD for *F. americana* at the *seedling* recruitment stage, despite the absence of notable CNDD at the *sapling* stage.

We do not know the mechanisms responsible for the neighborhood effects documented here, but our results do provide some clues. For instance, the two *Carya* species for which only negative conspecific effects were found (*C. glabra* and *C. ovalis*) are considered moderately shade‐tolerant, while the two species that were negatively affected by both conspecific and heterospecific adults (*C. cordiformis* and *C. tomentosa*) are considered intolerant of shade (Samuelson & Hogan, 2003). This presents the possibility that our results for *C. cordiformis* and *C. tomentosa* may be driven largely by a general reaction to light availability; however, in the case of *C. tomentosa*, the heterospecific effects were absent in the growth‐based point pattern analyses and less pronounced in the regressions (in terms of significance, standardized slope estimates, and partial *r* ^2^ values), suggesting the existence of a species‐specific mechanism (e.g., a specialist pathogen or insect pest). Similarly, the absence of significant heterospecific effects for *C. glabra* and *C*. *ovalis* implies that one or more species‐specific drivers may be operating. This same argument applies to *F. grandifolia*, a highly shade‐tolerant species (Tubbs & Houston, 1990) that exhibited no evidence of heterospecific inhibition. Ramage and Mangana (2017) also detected CNDD in this species and suggested that one or more common beech‐specific pests (e.g., *Grylloprociphilus imbricator* [beech blight aphid] and *Epifagus virginiana* [beechdrops, a parasitic plant]) could be responsible for depressed performance near adult conspecifics.

In addition to documenting neighborhood effects on sapling growth rates, our work provides a comparison among different analytical approaches. In particular, results from our size‐based point pattern analyses were poorly aligned with both of our growth‐based approaches, providing further evidence that analyses of static spatial patterns often fail to reveal underlying processes (e.g., variation in growth rates) (McIntire & Fajardo, 2009). When there is variation in ages of small trees, neighborhood effects on growth rates will not necessarily translate to spatial patterns in size classes, because growth rate and age may be confounded (Canham, 1990). Accordingly, we should not expect static spatial patterns to accurately reveal species‐specific effects of adult trees on juvenile growth rates unless the forest being studied contains a single even‐aged juvenile cohort below a mature, mixed‐species canopy. In this situation, species subject to, for instance, strong negative conspecific neighbor effects on sapling growth rates will also exhibit reduced clustering around conspecific adults as size class increases (e.g., 1‐ to 5‐cm DBH trees would be more clustered around conspecific adults than 5‐ to 10‐cm DBH trees). As a demonstration of this limitation, our regressions and growth‐based point pattern analyses both revealed a negative conspecific effect for *C. glabra*, but our size‐based point pattern analyses found that two different small stem size classes (1–5 cm DBH and 5–10 cm DBH) were equally clustered around conspecific adults. Taken together, these results suggest that small *C. glabra* stems close to conspecific adults may have a higher mean age than similarly sized small stems at greater distances. However, we did detect a signal of CNDD in the size‐based point pattern analyses for *C. tomentosa* (matching the regressions and growth‐based point pattern analyses), which could suggest: (1) very strong negative conspecific effects close to adult trees, leading to near total stagnation in growth, or (2) a major pulse of past recruitment such that most of the stems between 1 and 10 cm DBH are approximately the same age.

Despite the limitations of snapshot (i.e., single point in time) spatial patterns, several studies have drawn CNDD‐related inferences from analyses of these patterns. For instance, Johnson et al. (2014) subjected numerous tree species across three sites in the eastern United States (including our study site) to bivariate point pattern analyses. They found that small stems (1–12.7 cm DBH) of most species exhibited random patterns with respect to conspecific adults, but about one‐third of species were significantly over‐dispersed from adults, and suggested that Janzen‐Connell effects may explain that pattern. Unlike our analyses, they did not directly compare the clustering of multiple size classes around adult trees, but they did provide a figure (Fig. 3 in Johnson et al., 2014) that enables a rough visual comparison of the spatial distribution of 1‐ to 2.54‐cm and 2.54‐ to 12.7‐cm DBH stems around adults. Wiegand et al. (2007) also inferred Janzen‐Connell effects from a snapshot spatial analysis. Focusing on the most abundant species in a Sri Lankan forest dynamics plot, *Shorea congestiflora*, they found that recruits were much more dispersed than expected around adult trees. As with Johnson et al. (2014), they did not directly compare multiple size classes but did provide a figure (Fig. 5 in Wiegand et al., 2007) that shows the spatial distribution of both 1‐ to 5‐cm and 5‐ to 10‐cm DBH stems around adult conspecifics. In both cases (Johnson et al., 2014 and Wiegand et al., 2007), differences in clustering around conspecific adults, across small stem size classes, are not readily apparent from the figures provided. While these studies provide evidence of CNDD, they do not elucidate whether the observed patterns of overdispersion were influenced by conspecific effects on growth, as opposed to survival.

Preliminary analyses of our dataset (not shown) revealed very low mortality rates in 1‐ to 5‐cm DBH stems (the smallest mapped size class at our study site), precluding the possibility of mortality‐focused analyses. While it is possible that conspecific effects on sapling survival might have been detected with a much larger sample size, several previous studies have found that mortality beyond the seedling stage is largely unaffected by local conspecific density (Wills & Condit, 1999; Zhu et al. 2015a). Furthermore, prior research conducted at our study site found a mean age of 47 years for 5‐ to 10‐cm DBH trees, across a wide range of species (McGarvey et al., 2013), indicating that many species can survive for decades while growing at extremely slow rates (“oskars” sensu Silvertown, 1987). Collectively, these findings suggest that even severe suppression of growth rates will not necessarily lead to mortality, and that conspecific effects on growth rates alone have the potential to increase the local diversity of canopy trees.

Our findings provide further evidence that CNDD occurs in temperate forests, while also demonstrating that this process extends to sapling growth rates. In addition, our results raise the possibility that CNDD‐driven variation in growth rates could increase the local diversity of the forest canopy, even if there are no effects on survival, if large trees cause total or near total stagnation of proximate conspecific recruits. More broadly, if neighborhood effects often vary across life stages (e.g., seedling vs. sapling) and response metrics (e.g., survival vs. growth), the cumulative effects of CNDD may be more widespread and more complex than generally appreciated. Our different analytic approaches also illustrate the strengths and shortcomings of different methods. Integrating repeated measurements, point pattern analyses, and neighborhood models clearly reveals the problems with inferring process from snapshot spatial analyses. At the same time, by combining two approaches directly informed by growth rates, we can confidently assess interspecific variation in conspecific effects. However, point pattern analyses informed by growth rates require at least two censuses (or coring or destructive sampling) of large, fully mapped plots, and it is noteworthy that even in a relatively low diversity forest (compared to the tropics), most tree species were too rare for proper analysis. While the widespread occurrence of CNDD across latitudes and size classes is becoming more evident, thorough investigation of the interactions between CNDD and other factors will require expansive datasets resulting from long‐term, large‐scale data collection efforts.

## CONFLICT OF INTEREST

None declared.

## AUTHOR CONTRIBUTIONS

BR and DJ conceived the study and conducted the analyses. BR, DJ, and KC wrote the majority of the manuscript. EG, KA, NB, and WM led data collection efforts. All authors provided critical input to manuscript drafts and approved the final manuscript for publication.

## DATA ACCESSIBILITY

Full census data are available through the CTFS‐ForestGEO data repository (http://ctfs.si.edu/Public/plotdataaccess/), and 2008 census data are also published in Bourg et al. (2013; https://doi.org/10.1890/13-0010.1).
