# Supplementary material for: Sapling growth rates reveal conspecific negative density dependence in a temperate forest
Source: Ecol Evol. 2017 Aug 18;7(19):7661–71. doi: 10.1002/ece3.3298 (PMC5632615; doi:10.1002/ece3.3298)
Supplement: Supplementary file 1 [file ECE3-7-7661-s001.pdf]

Results from analyses with deer exclosure omitted

REGRESSIONS

Table S1. Sapling growth rate as a function of conspecific and heterospecific inverse distance-weighted basal area, **with the deer exclosure omitted.** A corresponding table with the deer exclosure included is provided in the main text (Table 2; see caption for additional details). Cells with brackets identify qualitative differences from Table 2 (in the main text). *L. tulipifera* could not be analyzed with the exclosure omitted because there was a very low number of saplings (5) outside of the exclosure.

Species	n	Conspecific effect				Heterospecific effect			
		P-value	Estimate	Partial r2	Mean con. IDW BA (m^2)	P-value	Estimate	Partial r2	Mean het. IDW BA (m^2)
Acer rubrum	35	0.466	0.048	0.023	0.002	0.207	0.083	0.068	0.265
Carya cordiformis *	80	**0.017**	-0.252	0.101	0.007	**0.006**	-0.180	0.132	0.236
Carya glabra *	336	**0.032**	-0.048	0.016	0.014	0.280	-0.023	0.003	0.235
Carya ovalis	33	**0.028**	-0.121	0.179	0.007	0.672	-0.021	0.007	0.281
Carya tomentosa *	262	[0.112]	-0.036	0.011	0.012	[0.440]	-0.017	0.003	0.242
Fagus grandifolia	44	**0.019**	-0.257	0.101	0.045	0.176	-0.127	0.042	0.226
Fraxinus americana *	56	0.197	0.106	0.042	0.003	0.359	-0.075	0.021	0.270
Liriodendron tulipifera	5	NA	NA	NA	NA	NA	NA	NA	NA
Nyssa sylvatica *	240	0.744	0.008	0.000	0.012	**[0.048]**	-0.049	0.019	0.233

Interpretation of differences between Table S1 (above) and Table 2 (main text):

The qualitative p-value differences for *C. tomentosa* (conspecific and heterospecific) do not appear to be due to ecological differences between the areas inside and outside of the exclosure. Instead, we believe the discrepancy arises from the fairly subtle effects in the full analysis (low standardized slope estimates and partial r^2 in Table 2), combined with the substantial reduction in sample size when the exclosure is omitted (368 vs. 262). In support of this assertion, both slope estimates are similar (but not as pronounced) in the reduced data set, as compared to the full analysis. In addition, the mean conspecific and heterospecific IDW BA values are nearly identical across both analyses, indicating that the relative abundance of conspecific and heterospecific canopy trees is not notably different within the exclosure. Finally, an analysis of the exclosure alone (results not shown) also yields an absence of significant effects. Since both components of the full set (inside and outside of the exclosure) return non-significant results, it is highly likely that the significant effects in the full analysis are primarily due to a larger sample size.

For *N. sylvatica*, an analysis of the exclosure alone (results not shown) yields a highly non-significant p-value for the heterospecific effect (p=0.991), despite similar mean conspecific and heterospecific IDW BA values, suggesting that the shift from a non-significant p-value (in the full analysis) to a barely significant p-value (in the analysis omitting the exclosure) may reflect a real ecological distinction.

Note that direct comparisons between the areas inside and outside of the exclosure are not universally reported because sample sizes within the exclosure are very small for most species.

POINT PATTERN ANALYSES (GROWTH-BASED)

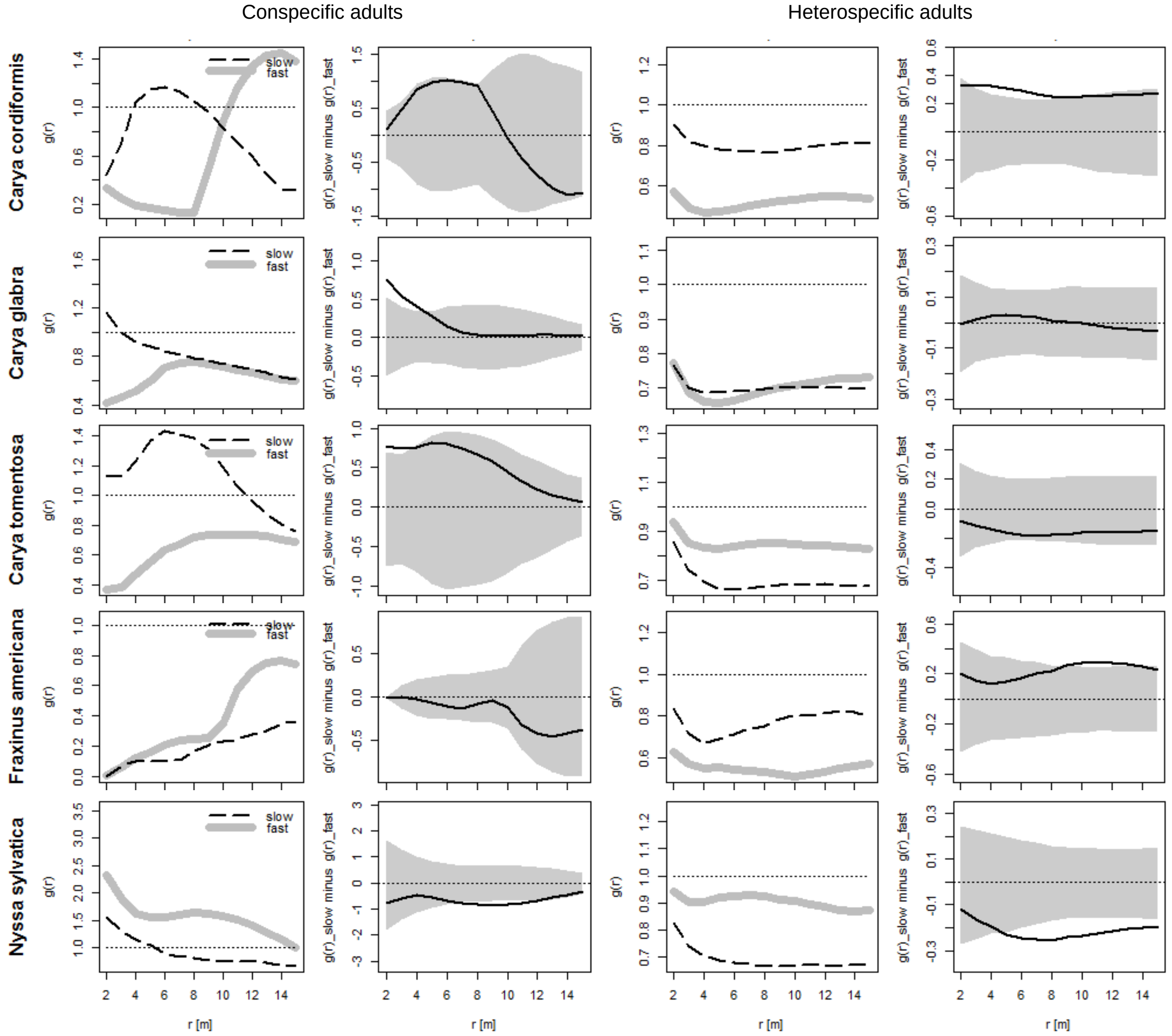

Fig. S1. Spatial patterns of two sapling categories (slow- and fast-growing) with respect to conspecific and heterospecific adult trees, **with deer exclosure omitted**. A corresponding figure with the deer exclosure included is provided in the main text (Fig. 1). Higher values represent increased clustering. The panels with confidence envelopes display slow values minus fast values; extensions above the envelope indicate that slow-growing saplings are more clustered than fast-growing saplings around adults (conspecific or heterospecific), and vice versa.

- ➔ The only qualitative difference between Fig. S1 (above) and Fig. 1 (main text) is that Fig. S1 indicates a tendency for slower growing *Nyssa sylvatica* saplings to be repelled from heterospecific adults (at intermediate and long distances), but Fig. 1 reveals no significant heterospecific effects for this species.

POINT PATTERN ANALYSES (SIZE-BASED)

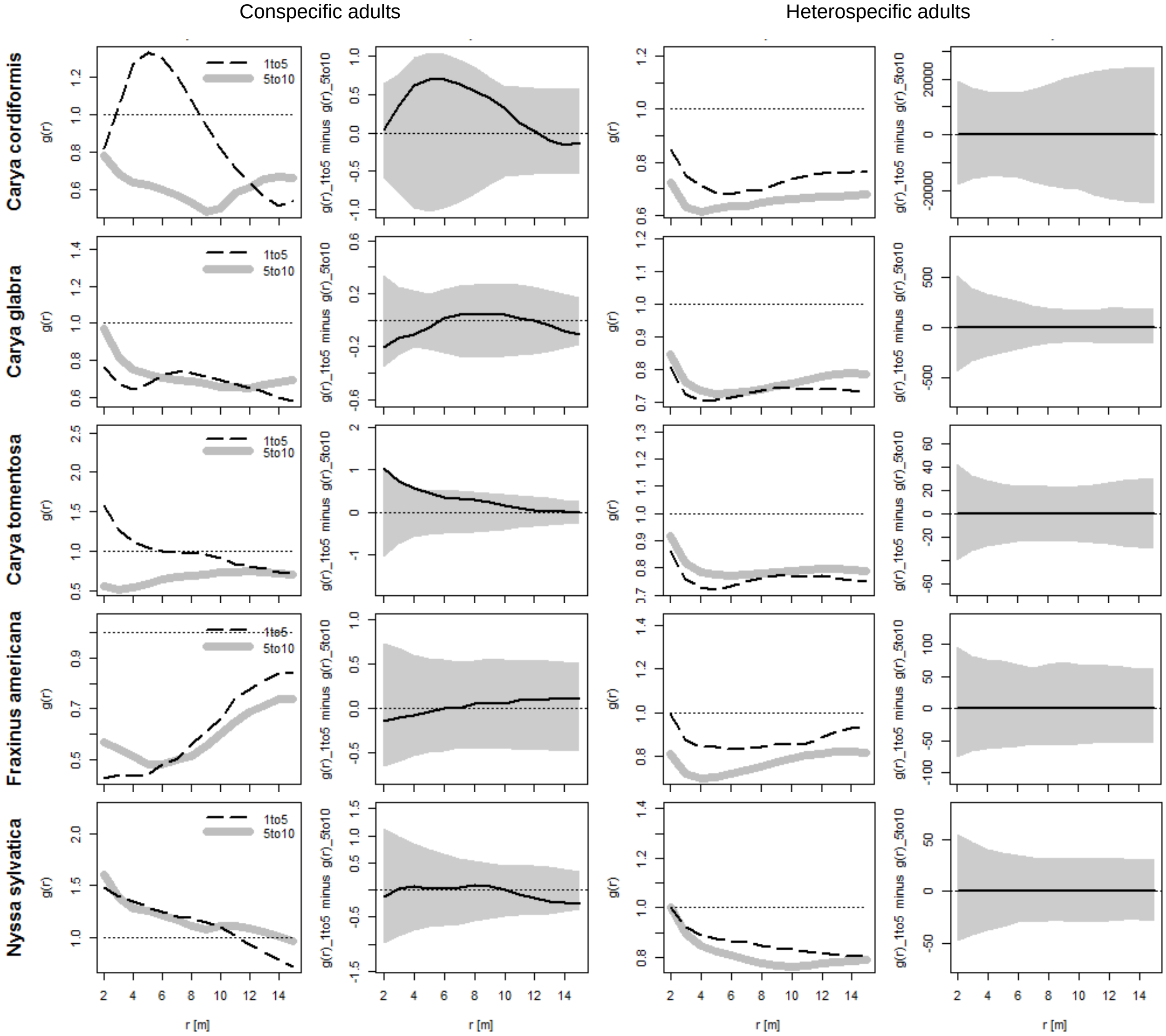

Fig. S2. Spatial patterns of two small stem categories (1-5 cm DBH [“saplings”] and 5-10 cm DBH) with respect to conspecific and heterospecific adult trees, **with deer exclosure omitted**. A corresponding figure with the deer exclosure included is provided in the main text (Fig. 2). Higher values represent increased clustering. The panels with confidence envelopes display “1to5” values minus “5to10” values; extensions above the envelope indicate that 1-5cm DBH stems are more clustered than 5-10cm DBH stems around adults (conspecific or heterospecific), and vice versa.

- ➔ The only qualitative difference between Fig. S2 (above) and Fig. 2 (main text) is that Fig. 2 indicates a tendency for smaller *Nyssa sylvatica* juveniles (1to5) to cluster more around conspecific adults, as compared to larger *N. sylvatica* juveniles (5to10), from distances of about 5 to 15 meters, but Fig. S2 reveals no significant effects for this species.

Congeneric effects among *Carya* species

We found mixed but limited support for con*generic* NDD among hickories (Table S2). Most pairwise comparisons were non-significant, but *C. glabra* had a negative effect on *C. tomentosa* and *C. ovalis* (which is sometimes considered a subspecies of *C. glabra*), and *C. tomentosa* negatively affected *C. glabra* and *C. cordiformis* (but the latter effect was borderline significant). When all *Carya* were pooled, only *C. ovalis* and *C. tomentosa* were negatively affected, matching the independent effects of *C. glabra*, and thus suggesting that *C. glabra* may be driving the overall pattern. No positive congeneric effects were detected. Overall, con*specific* NDD seems to be stronger than con*generic* NDD among hickories, suggesting that the underlying mechanisms (e.g. resource competition, shared natural enemies) are mostly species-specific. However, Zhu et al. (2015a) found – in a tropical forest – that negative effects beyond the species level (i.e. phylogenetic NDD) increased with life stage (and were only significant in size classes beyond the sapling stage), while conspecific NDD decreased with life stage. As such, it is possible that the relative importance of congeneric and conspecific NDD in hickories is dependent on the size class investigated.

Table S2. Congeneric effects among *Carya* species (sapling growth rate as a function of IDW BA). In the four leftmost results columns, each cell is from a separate model with one *Carya* species as the focal species and another singled out as a heterospecific species of interest (so that each can be evaluated independently, one by one). These models also included conspecific IDW BA and IDW BA for all other heterospecific species (pooled together), but results are only shown for the *Carya* species of interest. The three rightmost *columns* are all from the same model, but each *row* represents a separate model. All other details match those described in Table 2 in the main text (e.g. spatial structure was included when appropriate). "0" = non-significant effect; "(-)" = borderline negative effect [0.05<p<0.10]; "-" = negative effect [0.01<p<0.05]; "--" = strong negative effect [p<0.01]; "X" = conspecific effect not shown here (instead see rightmost columns and Table 2).

Effect of: / On:	*Carya cordi.*	*Carya glabra*	*Carya ovalis*	*Carya tomen.*	Consp	All other *Carya*	All other hetero
Carya cordiformis	X	0	0	(-)	--	0	--
Carya glabra	0	X	0	-	-	0	0
Carya ovalis	0	-	X	0	--	-	(-)
Carya tomentosa	0	-	0	X	0	-	0

Additional information is revealed via a comparison of Table S2 (above) and Table 2 (main text). After separating the *Carya* species from other heterospecific species, a negative conspecific signal remains for all hickory species except *C. tomentosa*; in some ways this change is surprising given the strong support for a conspecific effect for *C. tomentosa* in the simpler model (Table 2), but at the same time, the corresponding partial r^2 is very low (0.023). The loss of a negative conspecific signal occurs regardless of whether or not the residual spatial structure, which is borderline significant in this case, is included in the model. For *C. cordiformis*, the strong general heterospecific effect remains after separating out the other *Carya* species, but for *C. tomentosa*, it appears that the negative heterospecific effect was driven by other hickories.
